# THE FIT OF THE HAPA MODEL TO THE EXPERIENCE OF EXERCISING AFTER STROKE: A DEDUCTIVE CONTENT ANALYSIS

**DOI:** 10.1093/geroni/igac059.2463

**Published:** 2022-12-20

**Authors:** Amy Silva-Smith

**Affiliations:** University of Colorado at Colorado Springs, Colorado Springs, Colorado, United States

## Abstract

Sustained physical activity is recommended for secondary stroke prevention. Persons with stroke leave rehabilitation having learned exercises to reduce disability. However, once discharged, people may be on their own to navigate psychological, emotional, social, and physical challenges of maintaining those activities and starting new ones. The Health Action Process Approach (HAPA) provides a framework for understanding how self-efficacy differs depending on where a person is in the process of engaging in physical activity. The purpose of this study was to assess the qualitative fit of the HAPA model to the experience of exercise after discharge from formal stroke rehabilitation using a deductive (directed) content analysis approach. Interviews with 12 stroke survivors were analyzed deductively using the HAPA model concepts task self-efficacy, coping self-efficacy, and recovery self-efficacy to create the analysis matrix. In this sample, a period of psychological adjustment interfered with maintaining exercise and included anxiety, depression, embarrassment, and fear of falling that affected motivation and intention to exercise. Experiences with physical activity and exercise as a child and routines prior to the stroke were factors influencing task, coping, and recovery self-efficacy and ease of dealing with interruptions in exercise, including the discharge from formal rehabilitation. The findings support the qualitative fit of the HAPA model with the experience of exercise after having a stroke. A HAPA model framed intervention is being developed to support the transition from formal rehabilitation support to living in the community.

